# Concentration- and pH-Dependent Oligomerization of the Thrombin-Derived C-Terminal Peptide TCP-25

**DOI:** 10.3390/biom10111572

**Published:** 2020-11-19

**Authors:** Ganna Petruk, Jitka Petrlova, Firdaus Samsudin, Rita Del Giudice, Peter J. Bond, Artur Schmidtchen

**Affiliations:** 1 Division of Dermatology and Venereology, Department of Clinical Sciences, Lund University, 22241 Lund, Sweden; jitka.petrlova@med.lu.se (J.P.); artur.schmidtchen@med.lu.se (A.S.); 2 Bioinformatics Institute (BII), Agency for Science, Technology and Research (A*STAR), Singapore 138671, Singapore; mohdfbs@bii.a-star.edu.sg (F.S.); peterjb@bii.a-star.edu.sg (P.J.B.); 3 Research Centre for Biointerfaces, Department of Biomedical Sciences and Biofilms, Malmö University, 20506 Malmö, Sweden; rita.del-giudice@mau.se; 4 Department of Biological Sciences, National University of Singapore, Singapore 117543, Singapore; 5 Copenhagen Wound Healing Center, Bispebjerg Hospital, Department of Biomedical Sciences, University of Copenhagen, 2200 Copenhagen, Denmark; 6 Dermatology, Skane University Hospital, 22185 Lund, Sweden

**Keywords:** oligomerization, thrombin, peptide self-assembly, antimicrobial peptide, pH- and/or concentration-sensitive oligomerization, TCP-25

## Abstract

Peptide oligomerization dynamics affects peptide structure, activity, and pharmacodynamic properties. The thrombin C-terminal peptide, TCP-25 (GKYGFYTHVFRLKKWIQKVIDQFGE), is currently in preclinical development for improved wound healing and infection prevention. It exhibits turbidity when formulated at pH 7.4, particularly at concentrations of 0.3 mM or more. We used biochemical and biophysical approaches to explore whether the peptide self-associates and forms oligomers. The peptide showed a dose-dependent increase in turbidity as well as α-helical structure at pH 7.4, a phenomenon not observed at pH 5.0. By analyzing the intrinsic tryptophan fluorescence, we demonstrate that TCP-25 is more stable at high concentrations (0.3 mM) when exposed to high temperatures or a high concentration of denaturant agents, which is compatible with oligomer formation. The denaturation process was reversible above 100 µM of peptide. Dynamic light scattering demonstrated that TCP-25 oligomerization is sensitive to changes in pH, time, and temperature. Computational modeling with an active 18-mer region of TCP-25 showed that the peptide can form pH-dependent higher-order end-to-end oligomers and micelle-like structures, which is in agreement with the experimental data. Thus, TCP-25 exhibits pH- and temperature-dependent dynamic changes involving helical induction and reversible oligomerization, which explains the observed turbidity of the pharmacologically developed formulation.

## 1. Introduction

Each year, infectious diseases account for millions of deaths worldwide and incur tremendous health care costs [1]. Wound infections are significant medical problems that affect postoperative and burn wound patients, as well as patients with non-healing wounds. Furthermore, the increase in resistance to antibiotics is a global threat [1,2]. Therefore, there is an urgent need for innovative and effective treatments.

Antimicrobial peptides (AMPs) are important components of innate immunity that mount a rapid and broad-spectrum response toward both Gram-negative and Gram-positive bacteria, as well as fungi [3,4,5,6]. Moreover, many AMPs are multifunctional in that they also mediate various immunomodulatory roles. This has recently motivated a broader definition of host defense peptides (HDPs) for these members of the innate immune system [7,8,9,10]. From this perspective, drugs based on AMPs/HDPs have shown promise as alternatives to conventional antibiotics, and several peptides are currently in clinical development for various indications [11,12].

The thrombin-derived C-terminal peptide GKYGFYTHVFRLKKWIQKVIDQFGE, denoted as TCP-25, encompasses endogenous TCP sequences identified in human wounds. TCP-25 is antimicrobial and protects against bacterial sepsis and lipopolysaccharide (LPS)-mediated shock in experimental animal models [13,14,15]. At the molecular level, the peptide neutralizes bacterial LPS and specifically interferes with CD14 and TLR2/4-induced NF-κB activation in response to several microbe-derived agonists [14,16,17]. In addition, the peptide reduces inflammatory responses to intact bacteria during phagocytosis [18] and inhibits neutrophil responses to LPS in vitro and in vivo [19].

A hydrogel containing TCP-25 was effective against *Staphylococcus aureus*, *Pseudomonas aeruginosa*, and clinical bacterial isolates in vitro. Furthermore, it has been used to treat murine models of subcutaneous infections and porcine partial-thickness wound infections in vivo [20]. Cleavage fragments of TCP-25 are similar to those found in human wound fluid, so treatment with TCP-25 hydrogel resulted in a boosting of HDP levels that are normally present during wound healing. Dual-action anti-inflammatory and antibacterial peptide-functionalized hydrogel also hastened the healing of infected wounds in pigs, thus making TCP-25 hydrogel a promising treatment approach for wound healing [20].

Preclinical studies were conducted using a formulation based on hydroxyethylcellulose, glycerol, and Tris-buffer at pH 7.4, and the results showed turbidity of the hydrogel, particularly when using the peptide at a high concentration (0.3 mM) [20]. Thus, although a therapeutic efficacy of the TCP-25 hydrogel was demonstrated, it still remained to be explored whether the observed turbidity was dependent on oligomer formation, and if so, whether it affected peptide structure, activity, as well as stability. With this as background, we therefore set out to investigate the mechanisms underlying the observed turbidity of TCP-25. We show that at pH 7.4, the peptide undergoes a dose-dependent increase in α-helical structure. This helical induction is indicative of self-interactions and is not observed at pH 5.0. Intrinsic tryptophan fluorescence shows that TCP-25 is more stable at a high concentration (0.3 mM) when exposed to high temperatures or a high concentration of denaturant agents, which is compatible with oligomer formation. Moreover, analysis by dynamic light scattering (DLS) demonstrates that the oligomerization of TCP-25 is highly dynamic and depends on pH, time, and temperature. Finally, molecular docking and computational multiscale simulations using an active 18-mer region of TCP-25 support the data and show how the peptide may form pH-dependent higher-order end-to-end oligomers and micelle-like structures.

## 2. Materials and Methods

### 2.1. Peptide

The thrombin-derived peptide TCP-25 (GKYGFYTHVFRLKKWIQKVIDQFGE) was synthesized by AmbioPharm, Inc. (North Augusta, SC, USA). The purity was over 95%, which was confirmed by mass spectral analysis (MALDI-TOF Voyager, Applied Biosystems, Watertown, MA, USA).

### 2.2. Turbidity Assay

TCP-25 was resuspended in 10 mM Tris at pH 7.4 or in 10 mM NaOAc at pH 5 and 5.8 at increasing concentrations (10–300 μM) and incubated for 1 h at room temperature (RT). Then, the turbidity was monitored by measuring the absorbance and transmittance at 405 nm [21] using a DU^®^ 800 UV/Visible Spectrophotometer (Beckman Coulter^TM^, Fullerton, CA, USA).

### 2.3. Electrophoresis and Western Blot

TCP-25 was resuspended in 10 mM Tris at pH 7.4 or in 10 mM NaOAc at pH 5 and 5.8 at a concentration of 1 mM. Next, 30 μL of the respective samples were centrifuged at 14,000× *g* for 20 min, and then 10 μL of the supernatant and the complete pellet were loaded on a 10–20% Novex Tricine pre-cast gel (Invitrogen, Carlsbad, CA, USA). Electrophoresis was performed at 100 V for 100 min. The gel was stained using Coomassie Brilliant blue (Invitrogen, Rockford, IL, USA), and images were obtained using a Gel Doc Imager (Bio-Rad Laboratories, Hercules, CA, USA).

To analyze the oligomerization, a range of TCP-25 concentrations (10–300 μM in 10 μL) was analyzed by BN-PAGE (NativePAGE Bis-Tris Gels System 4–16%, Invitrogen, Carlsbad, CA, USA) according to the manufacturer’s instructions. For western blotting, the material was subsequently transferred to a PVDF membrane using a Trans-Blot Turbo system (Bio-Rad, Laboratories, Hercules, CA, USA). TCP-25 was detected using polyclonal rabbit antibodies against the C-terminal thrombin epitope VFR17 (VFRLKKWIQKVIDQFGE; diluted 1:1000, Innovagen AB, Lund, Sweden), followed by porcine anti-rabbit HRP conjugated antibodies (1:1000, Dako, Glostrup, Denmark). The peptide was visualized by incubating the membrane with SuperSignal West Pico Chemiluminescent Substrate (Thermo Scientific, Rockford, IL, USA) for 5 min, followed by detection using a ChemiDoc XRS Imager (Bio-Rad Laboratories, Hercules, CA, USA).

### 2.4. Circular Dichroism Spectroscopy

Circular dichroism (CD) was used to analyze the changes in the secondary structure of TCP-25 at different concentrations (10–300 μM) and in different buffer systems (10 mM Tris at pH 7.4, 10 mM NaOAc at pH 5 and 5.8). The measurements were performed on a Jasco J-810 spectropolarimeter (Jasco, Tokyo, Japan) equipped with a Jasco CDF-426S Peltier set to 25 °C. Quartz cuvettes (0.1 and 0.2 cm) (Hellma, GmbH & Co, KG, Müllheim, Germany) were used for TCP-25 concentrations of 100–300 μM and 10–30 μM, respectively. The spectra were recorded at 190–260 nm (scan speed: 20 nm min^−1^) as an average of 5 measurements. Raw spectra were corrected for buffer contribution and converted to the mean residue ellipticity, θ (mdeg cm^2^ dmol^−1^). The secondary structure was estimated according to the equation reported by Morrissette et al. [22].

### 2.5. Transmission Electron Microscopy

The oligomers of TCP-25 were visualized by transmission electron microscopy (TEM) (Jeol Jem 1230; Jeol Ltd., Tokyo, Japan) in combination with negative staining. In particular, 10 and 300 μM TCP-25 dissolved in 10 mM Tris at pH 7.4 or in 10 mM NaOAc at pH 5 were analyzed. After dissolving, 5 μL of each sample were adsorbed onto carbon-coated grids (Copper mesh, 400) for 60 s and stained with 7 μL of 2% uranyl acetate for 30 s. The grids were rendered hydrophilic via glow discharge at low air pressure [23]. Analysis was done on 10 view fields (magnification 4200×) of the mounted samples on the grid (pitch 62 μm) from three independent experiments.

### 2.6. Chemical Crosslinking

Twenty μL of 1 mM TCP-25 dissolved in 20 mM HEPES were incubated with increasing concentrations of BS^3^ (from 18–580 μM) for 30 min at RT. The crosslinking reaction was terminated by the addition of 1 μL of 1 M Tris pH 7.4. The oligomers that formed were analyzed on a 10–20% Novex Tricine pre-cast gel (Invitrogen, Carlsbad, CA, USA), followed by Coomassie staining as described above. 

### 2.7. High-Pressure Liquid Chromatography (HPLC)

Peptide samples cross-linked with 145 and 580 μM of BS^3^ were further characterized by reverse-phase chromatography on a Phenomenex Kinetex C18-column (50 × 2.1 mm 2.6 μM, 100 Å pore size, Torrance, CA, USA) using the Agilent 1260 Infinity System. The column was equilibrated using 95% of buffer A containing 0.25% of Trifluoroacetic acid (TFA) in MilliQ and 5% of buffer B containing 0.25% of TFA in acetonitrile. The peptide with or without crosslinker was dissolved in buffer A (1:3), and 3 μg were injected onto the system. The elution profile was monitored during the gradient (35% of B at 5 min, 45% at 10 min), and the spectrum at 215 nm was recorded. The flow rate of the column was 0.5 mL min^−1^, and all runs were performed at RT.

### 2.8. Thermal and Chemical Denaturation

Thermal and chemical denaturation was analyzed by recording protein emission fluorescence spectra at 300–450 nm following excitation at 280 nm. The intrinsic fluorescence of 10 and 300 μM TCP-25 dissolved in 10 mM Tris at pH 7.4 or 10 mM NaOAc at pH 5, respectively, was measured in a 10-mm quartz cuvette. The measurements were done using a Jasco J-810 spectropolarimeter equipped with an FMO-427S fluorescence module at a scan rate of 200 nm min^−1^ with a 2-nm slit width. Thermal denaturation was induced by increasing the temperature from 20 to 100 °C with increments of 2 °C at 20–30 °C and with increments of 5 °C at 30–100 °C. The peptide was incubated at the desired temperature for 10 min before taking the measurements.

T_m_ was determined by fitting the maximum emission fluorescence as a function of increasing temperature. Chemical denaturation was performed by incubating the peptide at 4 °C for 24 h with increasing concentrations (0–5 M) of urea or guanidinium chloride (Gnd-HCl) before measuring the intrinsic fluorescence. C_m_ was then calculated with the fluorescence ratio (F337/F350) as a function of the concentration of the chemical agent [24]. The results are expressed as the average of three independent experiments ± SEM.

### 2.9. Dynamic Light Scattering

The size of TCP-25 oligomers and their relative concentrations in solution were determined using Multi-angle dynamic light scattering (MADLS^®^) on a Zetasizer Ultra (Malvern Panalytical, Ltd, Malvern, UK) using a quartz cuvette (ZEN2112) with a final volume of 75 μL. The peptide was dissolved in 10 mM Tris at pH 7.4 or in 10 mM NaOAc at pH 5 at a concentration of 300 μM immediately before the first data acquisition. The oligomerization rate was monitored at different time points (0–24 h and after 1 week) and after storage at different temperatures (RT, 4 and −20 °C).

For the peptide stored at −20 °C, time 0 refers to the reading immediately after melting. All reads were taken at 25 °C. Data were processed using the software Zetasizer Ultra-Pro ZS Xplorer version 1.31. Based on the hydrodynamic diameters, the peptide oligomers/aggregates were classified into 4 different families: small (0.4–5 nm), medium (20–150 nm), large (200–950 nm), and giant (1–5×10^3^ nm). For each sample, spectra were recorded three times with 11 sub-runs using the multimodal mode. In the graphs, the concentration of the oligomers belonging to different families is reported as the average ± SD.

### 2.10. Molecular Modeling and Simulation

To model the oligomerization of HVF18 peptides, two structural inputs were used: (i) the nuclear magnetic resonance (NMR) structure of LPS-bound HVF18 (PDB: 5Z5X) [17] and (ii) a model of HVF18 peptide constructed as an ideal alpha-helix using Modeller version 9.21 [25]. The best model was chosen from 10 outputs based on the lowest discreet optimized protein energy (DOPE) score [26] with no residues outside the favored region in Ramachandran space [27]. The protein-protein docking web server, ClusPro, was then used to model dimers of HVF18 peptides [28]. A representative structure from the most populated clusters was chosen for simulation. The HVF18 dimer was parameterized using the CHARMM36 force field [29] and solvated with TIP3P water molecules containing 0.15 M NaCl salt. All ionizable residues were assigned their default charge state at the relevant pH, as supported by pKa calculations performed using PROPKA3 [30]. Energy minimization and equilibration simulations were performed using the CHARMM-GUI standard protocols [31].

A 200-ns production simulation was conducted using GROMACS 2018 [32]. The Nosé–Hoover thermostat [33,34] and Parrinello–Rahman barostat [35] were used to maintain the temperature and pressure at 298 K and 1 atm, respectively. Electrostatic interactions were calculated using the smooth particle mesh Ewald (PME) method [36] with a real-space cutoff of 1.2 nm, whereas van der Waals interactions were cut off at 1.2 nm with the force-switch smoothing function applied between 1.0 and 1.2 nm. All covalent bonds with hydrogen atoms were constrained using the LINear Constraint Solver (LINCS) algorithm [37], and an integration time step of 2 fs was used.

The structure of the HVF18 dimer at the end of the simulation was then used to build higher-order multimers using ClusPro. Representative structures from the most populated clusters from these docking simulations were selected, and a 200-ns simulation was performed using the same protocols described above to examine their stability. To mimic low-pH conditions, the histidine residue on the N-terminus, whose predicted pKa value was 6.34, was protonated. For coarse-grained (CG) self-assembly simulations, the HVF18 ideal helix homology model was converted to a CG representation using the MARTINI 2.2 force field [38], and an elastic network model, ElNeDyn, was applied to maintain helicity [39]. Two copies of the HVF18 models were placed 4 nm apart and solvated with standard MARTINI water particles and 0.15 M NaCl.

Energy minimization and equilibration simulations were performed following the CHARMM-GUI standard protocols [8]. Five independent 1-µs simulations were performed to allow the formation of an HVF18 dimer. The V-rescale thermostat was used to maintain the temperature at 298 K [40], while the Parrinello–Rahman barostat was used to maintain the pressure at 1 atm [35]. Electrostatic interactions were described using the reaction field method, whereas van der Waals interactions were computed using the potential shift Verlet scheme, both with a short-range cutoff of 1.1 nm. An integration time step of 10 fs was used. The structures of the HVF18 dimer at the end of the simulations were subsequently converted to an atomic representation using the CHARMM-GUI All-Atom Converter [18,41]. A representative structure was then used for a 200-ns atomistic simulation using the protocols described above.

### 2.11. Statistical Analysis

All the experiments were performed at least 3 times except for DLS, which was repeated 2 times. The results are presented as the means ± SD or SEM as indicated. The data were analyzed using GraphPad Prism (GraphPad Software, Inc., San Diego, CA, USA), and * indicates *P* < 0.05. P values were determined using one-way ANOVA with Dunnett’s multiple comparison test.

## 3. Results

### 3.1. Relationship between Turbidity and Oligomerization/Aggregation of TCP-25 

**Figure 1:**
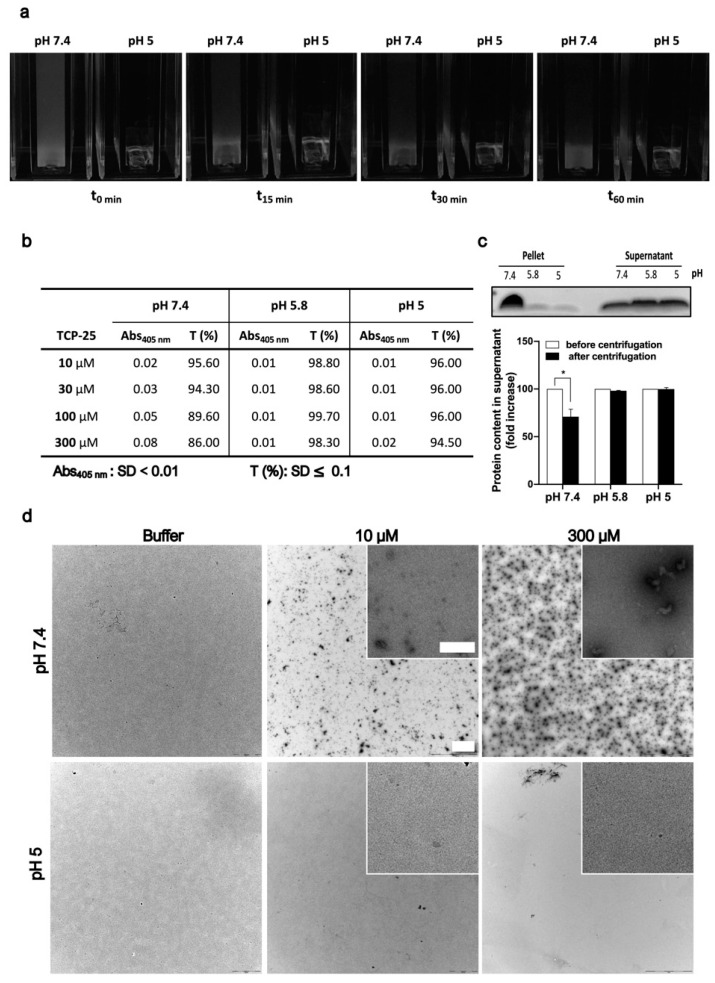
Effects of pH and concentration on TCP-25 oligomerization. (**a**) Representative pictures of cuvettes containing 300 µM TCP-25 dissolved in 10 mM Tris at pH 7.4 or 10 mM NaOAc at pH 5 immediately after storage at 4 °C (t_0_ min) and indicated time points after incubation at RT. (**b**) Absorbance and transmittance values at 405 nm for 10-300 µM TCP-25 dissolved in 10 mM Tris at pH 7.4 or in 10 mM NaOAc at pH 5.8 and 5. (**c**) SDS-PAGE analysis of pellets and supernatants from the centrifugation of 300 µM TCP-25 dissolved in 10 mM Tris at pH 7.4 or in 10 mM NaOAc at pH 5.8 or 5.0. The graph shows the TCP-25 concentration ± SD after centrifugation. (**d**) TEM images illustrating that oligomerization is pH and concentration dependent. TCP-25 was dissolved in pH 7.4 and 5.0 buffers at the indicated concentrations and analyzed by TEM. Scale bar in the 40× and 100× (insert) magnification window represents 2.5 and 0.25 µm, respectively. All experiments were performed 3 times (*n* = 3), and * indicates *p* < 0.05. *p* value was determined using one-way ANOVA with Dunnett’s multiple comparison test.

TCP-25 is a 3-kDa C-terminal thrombin peptide characterized by antimicrobial and anti-inflammatory activity in vitro and in vivo [14,16,18,20]. Currently, it is in the preclinical development stage. Solutions of 300 µM TCP-25 yielded a turbid appearance at pH 7.4, in contrast to pH 5, where the solution was markedly less turbid (Figure 1a). These observations prompted further investigations on the dependence of this phenomenon on the concentration and pH of TCP-25. Therefore, we analyzed the absorbance at 405 nm and the relative transmittance of TCP-25 at pH 5-7.4 and different concentrations. The results are summarized in Figure 1b and indicate that both the absorbance and transmittance change at pH 7.4 as the concentration of TCP-25 increases. These findings are compatible with the observed turbidity changes.

Considering the amphipathic nature of TCP-25, we hypothesized that the increase in turbidity could be dependent on the formation of oligomers or aggregates. To study this in more detail, we dissolved TCP-25 at a concentration of 300 μM in 10 mM Tris at pH 7.4 or in 10 mM NaOAc at pH 5.8 or 5.0. We centrifuged the samples and analyzed both the supernatant and the pellet by SDS-PAGE. Figure 1c illustrates that TCP-25 was indeed detected in higher amounts in the pellet obtained from the sample at pH 7.4.

Next, to explore whether the aggregated TCP-25 could be redissolved, we resuspended the pellet from the pH 7.4 sample in 10 mM Tris at pH 7.4 or in 10 mM NaOAc at pH 5.8 or 5. The results showed the same repartition in the pellet and supernatant after centrifugation as with the freshly prepared TCP-25 (Figure S1a), which indicates the reversibility of the observed oligomerization/aggregation of TCP-25. TEM was employed to visualize the oligomers/aggregates, which revealed multiple TCP-25 aggregates in Tris-buffer at pH 7.4, particularly at 300 µM (Figure 1d). This contrasted with the findings in NaOAc buffer at pH 5.0, where fewer aggregates where observed.

### 3.2. Structural Changes of TCP-25 Oligomers and Their Organization

**Figure 2:**
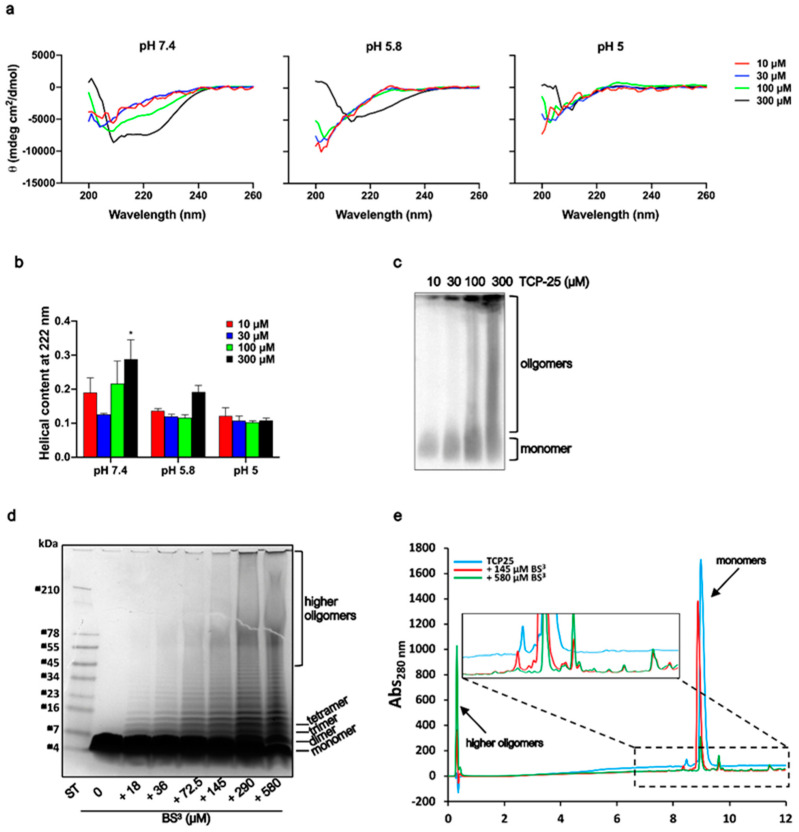
Structural analyses of TCP-25 oligomers. (**a**) Representative far-UV CD spectra of TCP-25 dissolved at 10, 30, 100 and 300 µM in 10 mM Tris at pH 7.4 or in 10 mM NaOAc at pH 5.8 and 5.0. All the spectra were acquired at 25 °C. The change in secondary structure was affected by pH and TCP-25 concentration (*n* = 3). (**b**) α-helical content ± SD calculated from CD spectra obtained at 222 nm. A significant increase in α-helical content was observed for 300 µM TCP-25 in 10 mM Tris at pH 7.4. * indicates *p* < 0.05 using one-way ANOVA with Dunnett’s multiple comparison test (*n* = 3). (**c**) Separation on 4–16% (*w*/*v*) BN-PAGE followed by western blot analysis shows an increased oligomerization of TCP-25 at higher concentrations. One representative image of 3 independent experiments is shown (*n* = 3). (**d**) TCP-25 was cross-linked with different concentration of BS^3^ for 30 min and then analyzed on 16.5% Tris-Tricine gel followed by Coomassie staining. Increased concentration of crosslinker yielded TCP-25 oligomers of higher molecular weights. One representative image of 3 independent experiments is shown (*n* = 3). (**e**) Reverse-phase C18 chromatography of TCP-25 in the absence (blue line) or in the presence of 145 µM (red line) or 540 µM (green line) BS^3^ showing an alteration in the elution profiles.

Peptide oligomerization can induce alterations in peptide secondary structure [42]. We used CD to observe whether TCP-25 secondary structure was affected by its oligomerization at pH 7.4 (Figure 2a and Figure S1b). TCP-25 was dissolved at different concentrations in 10 mM Tris at pH 7.4 or in 10 mM NaOAc at pH 5.8 or pH 5. As shown in Figure 2b, the peptide displayed a concentration-dependent increase in helicity at pH 7.4 with a dominant α-helical structure recorded at the highest concentration of 300 μM TCP-25. No significant concentration-dependent structural changes were observed in NaOAc at either pH 5.8 or 5.0, although an increase in helicity at pH 5.8 was noted for the peptide at 300 μM.

Given the propensity of TCP-25 to oligomerize in a concentration-dependent manner at pH 7.4, we decided to further analyze which oligomeric species were formed. For this purpose, freshly dissolved TCP-25 (10–300 μM) in 10 mM Tris at pH 7.4 was subjected to 4–16% (*w*/*v*) Blue native (BN)-PAGE. As shown in Figure 2c, TCP-25 formed a wide range of oligomers, and parts of the material did not enter the gel, indicating large oligomers or aggregates (bigger than 1500 kDa). To further characterize these oligomers, TCP-25 was chemically cross-linked with BS^3^.

As shown in Figure 2d, TCP-25 formed a broad spectrum of oligomers in agreement with the previous results. RP-HPLC analysis on a C18 column confirmed the presence of oligomers. When TCP-25 was cross-linked with BS^3^ at 580 μM, it yielded higher-order oligomers that eluted early in the gradient (Figure 2e). We also observed that the concentration-dependent oligomerization was reversible. Indeed, the CD spectra for the peptide at the same concentration were perfectly overlapping when TCP-25 was dissolved at 1 mM in 10 mM Tris at pH 7.4 and then diluted to 10 or 300 μM or when it was directly dissolved at these specific concentrations (Figure S1c).

### 3.3. Effects of Oligomerization on T_m_ and C_m_

**Figure 3:**
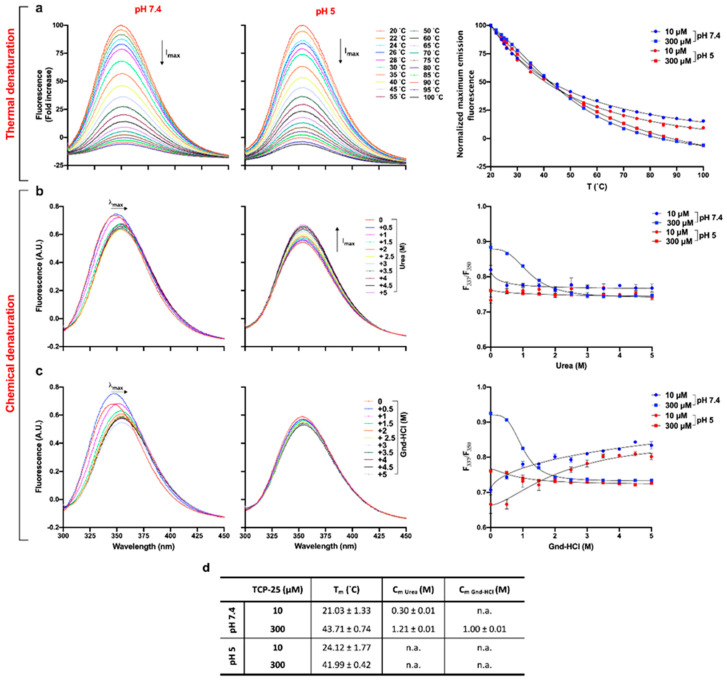
Thermal and chemical denaturation of TCP-25. TCP-25 (10 and 300 µM) in 10 mM Tris at pH 7.4 or in 10 mM NaOAc at pH 5.0 was denatured by increasing temperature (**a**) or by addition of increasing amounts of urea (**b**) or Gdn-HCl (**c**). The unfolding process was analyzed by recording the emission spectra between 300 and 450 nm upon excitation at 280 nm. Representative emission spectra are shown for 300 µM TCP-25 dissolved at pH 7.4 or 5.0 with different denaturing methods (*n* = 3). On the right, the denaturation curves are reported. In the case of thermal denaturation, data were obtained by fitting the normalized maximum emission fluorescence as a function of the temperature. For the chemical denaturation, results were obtained using the fluorescence ratio (F_337_/F_350_) as a function of the concentration of the chemical agent. Each data point represents the mean ± SEM (*n* = 3). (**d**) Table showing T_m_ and C_m_ ± SEM calculated from the denaturation curves obtained from 3 independent experiments done in duplicate (*n* = 3). → indicates shift in the maximum fluorescence intensity (λ_max_); ↑I_max_ and ↓I_max_ indicate increase and decrease in maximum fluorescence intensity, respectively.

The denaturation midpoint of a protein is defined as the temperature (T_m_) or concentration of denaturant (C_m_) at which both the folded and unfolded states are equally populated at equilibrium [43]. These parameters are expected to be altered in an oligomerized state [44]. Therefore, we next employed thermal and chemical denaturation assays to investigate the potential changes of T_m_ and C_m_ induced by oligomerization of TCP-25. The peptide was dissolved in 10 mM Tris at pH 7.4 or in 10 mM NaOAc at pH 5 at 10 and 300 μM. It was then subjected to thermal denaturation by increasing the temperature from 20 to 100 °C. Figure 3a shows representative fluorescence spectra for 300 μM TCP-25 dissolved at pH 7.4 and 5.0. As expected, at both pH values, the intrinsic fluorescence of the peptide decreased as the temperature increased. The same results were obtained for 10 μM TCP-25 at both pH 7.4 and 5.0 (Figure S2a).

T_m_ was determined by fitting the normalized maximum emission fluorescence as a function of the temperature (Figure 3a, left panel). We found that T_m_ was more affected by the concentration than pH changes (Figure 3d), which is compatible with the observed oligomerization of TCP-25 at higher concentrations. To determine C_m_, we used two different denaturant agents: urea (uncharged) and a salt of guanidine hydrochloride (Gnd-HCl). The latter is generally used when analyzing the specific contributions of hydrophobic or nonionic interactions to protein stability [45]. Therefore, we dissolved TCP-25 in the same conditions as above and incubated the peptide with increasing concentrations of urea or Gnd-HCl overnight before analysis. The results obtained for TCP-25 in the presence of both chemical agents are shown in Figure 3b–c.

Analysis of the fluorescence spectra obtained for the peptide dissolved at pH 7.4 at 300 μM showed that both urea and Gnd-HCl caused a red-shift in the maximum emission wavelength (λ_max_). This is indicative of a change in the solvent exposure of the tyrosine and tryptophan residues in TCP-25 (Figure 3b–c, left panels). Moreover, the unfolding induced by Gdn-HCl exhibited two transition phases. The first phase of the denaturation was characterized by an increase in fluorescence intensity and a small red-shift of λ_max_ in the sample with 0.5 M Gdn-HCl. This indicates the formation of an intermediate that has a higher fluorescence quantum yield than TCP-25 alone in the absence of the denaturing agent. When increasing the concentration of the Gdn-HCl up to 5 M, there was a decrease of fluorescence and a consistent red-shift in λ_max_ from 347 to 355 nm, which indicates the second phase of denaturation.

Completely different behavior was found for 300 μM TCP-25 dissolved at lower pH (Figure 3b–c, middle panels). Indeed, the λ_max_ of TCP-25 without any denaturant was already equal to 354 nm, which indicated that the Trp residues were already exposed to the polar environment. Moreover, in the case of denaturation by urea, a consistent increment in fluorescence intensity was also recorded, indicating that the species present in the solution were characterized by higher fluorescence quantum yield than the native form of TCP-25. The results for 10 μM TCP-25 dissolved at pH 7.4 and 5 showed a similar denaturation profile independently of the chemical agent used (Figure S2b–c). Furthermore, in all cases, we found an increase in fluorescence intensity and an evident blue-shift of λ_max_ of TCP-25 denatured with Gnd-HCl, which is an indication of lower exposure of Trp and Tyr to the solvent.

C_m_ was calculated for the two denaturants, and the I_337_/I_350_ ratio is reported as a function of the concentration of the chemical agent (Figure 3b–c, right panels), as summarized in the table in Figure 3d. In the presence of urea, C_m_ was much lower for 10 μM than for 300 μM TCP-25, which indicates that the observed concentration-dependent oligomerization of TCP-25 protects it from denaturation. In the case of Gnd-HCl denaturation, it was possible to determine C_m_ for only the 300 μM TCP-25 sample. It was not possible to determine C_m_ for the peptide dissolved at pH 5 under any denaturing conditions. This indicated that in its native state, the peptide is already largely unstructured, which is consistent with the CD data (Figure 2a). Altogether, the results from the thermal and chemical denaturation experiments are indicative of peptide aggregation/oligomerization.

### 3.4. Reversibility of Thermal Denaturation of TCP-25

**Figure 4:**
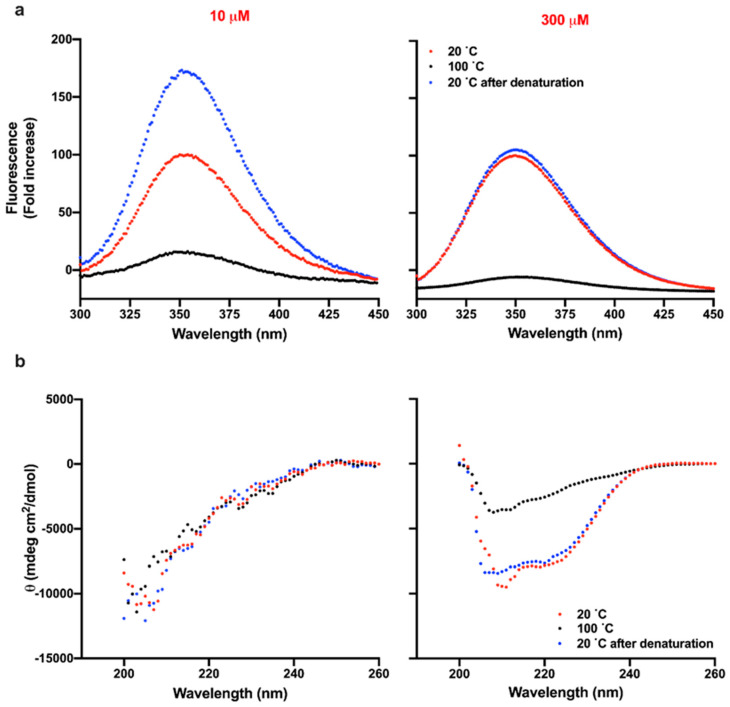
Reversibility of thermal denaturation of TCP-25 at pH 7.4. 10 and 300 µM TCP-25 in 10 mM Tris at pH 7.4 were denatured by exposing the peptide to 100 °C and bringing the temperature back to 20 °C. The re-folding was analyzed by recording the intrinsic fluorescence of the peptide (**a**) or secondary structure (**b**). The spectra were collected at 20 (red), 100 (black), and 20 °C after denaturation at 100 °C (blue). Each graph is a representative result of 3 independent experiments (*n* = 3).

Thermal unfolding of a protein is generally characterized by irreversible aggregation. To investigate whether this was also the case for TCP-25, we analyzed the structural changes of the peptide before and after denaturation at 100 °C. First, we compared the intrinsic fluorescence spectra of TCP-25 at 10 and 300 μM in 10 mM Tris at pH 7.4 or in 10 mM NaOAc at pH 5 at 20 °C before and after denaturation. As shown in the left panel of Figure 4a, the fluorescence of 10 μM TCP-25 dissolved at pH 7.4 increased around 1.75-fold upon denaturation in comparison to the non-denatured peptide. Moreover, λ_max_ was blue-shifted, which is indicative of aggregation of the peptide. Similar results were obtained for the peptide dissolved at pH 5 (Figure S3a).

Completely different behavior was observed in the case of TCP-25 dissolved at the same pH but at higher concentration. Indeed, the intrinsic fluorescence of TCP-25 at 20 °C was the same before and after exposing the peptide to 100 °C, suggesting reversibility (Figure 4a and Figure S3a). Slightly higher fluorescence and blue-shift of λ_max_ were found for 300 μM TCP-25 at pH 5 after the denaturation process, which is compatible with a less oligomerized peptide at the initiation of the experiment (Figure S3a).

To further confirm the reversibility of the thermal denaturation process, we analyzed the secondary structure of TCP-25 using CD before and after exposing the peptide to 100 °C. Figure 4b shows the results for the peptide dissolved at 10 and 300 μM in 10 mM Tris at pH 7.4. The conformation of the peptide was unstructured at low concentrations (left panel), but similar helical spectra were obtained before and after denaturation of TCP-25 at 300 μM, demonstrating reversibility of the denaturation (right panel). The data for 10 and 300 μM TCP-25 dissolved at pH 5 are reported in Figure S3b and confirm the reversibility of denaturation at higher concentrations.

### 3.5. Size of Oligomers as a Function of Temperature and pH

**Figure 5:**
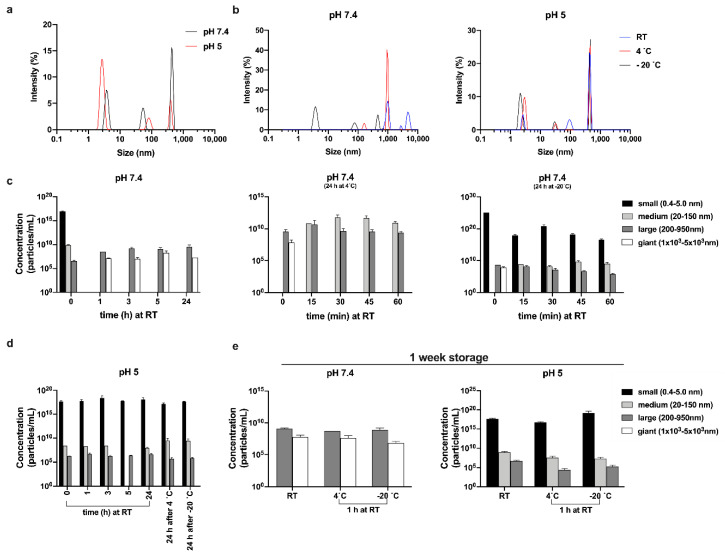
Size of oligomers and their distribution. (**a**,**b**) Representative size distribution by intensity of 300 µM TCP-25 in 10 mM Tris at pH 7.4 or in 10 mM NaOAc at pH 5.0. Sizes of oligomers at pH 7.4 (**c**,**e**) and pH 5.0 (**d**,**e**), and their distributions after storage at RT, 4, or –20 °C for up to 24 h (**c**,**d**) or after 1 week of storage (**e**). Oligomers were classified into 4 families: small (0.4–5 nm, black bars), medium (20–150 nm, light gray bars), large (200–950 nm, dark gray bars), and giant (1×10^3^ – 5×10^3^ nm, white bars). For each sample, spectra were recorded three times with 11 sub-runs using the multimodal mode. In the graphs, the concentrations of the oligomers belonging to different families are reported as the average ± SD (*n* = 2).

We employed DLS to gain further insights into the size of the oligomers and their relative distributions. TCP-25 was dissolved at 300 μM in 10 mM Tris at pH 7.4 or 10 mM NaOAc at pH 5 immediately before the first measurement. Figure 5a and Figure S4a show the results for the peptide at both pH values. The Z-average (mean particle size) was found to be higher for TCP-25 dissolved at pH 7.4 than at pH 5, indicating that the peptide forms bigger oligomers at pH 7.4. The polydispersity index (Pdi) was very high (0.68) in both cases, which is consistent with multiple species detected in the solution (for Pdi <0.1, the sample can be considered to be monodispersed [46]).

We next investigated the temperature dependence of the oligomerization process. The results in Figure 5b and Figure S4b were obtained after the analysis of TCP-25 stored for 24 h at RT, 4, and –20 °C. Much higher decay time and intensity were observed for the peptide at pH 7.4 for all storage conditions with respect to the peptide at pH 5, indicating an increase in the size of TCP-25 oligomers. Indeed, at pH 5, we found only a moderate rise of intensity with the decrease of temperature since low temperature generally promotes hydrophobic interactions [47]. Figure 5c–e show the size distribution of the oligomers and their concentrations in solution. For the freshly prepared sample (indicated as time 0 in the graphs), we detected particles spanning a broad range of hydrodynamic diameters. Therefore, we classified them into 3 families: small (0.4–5 nm), medium (20–150 nm), and large (200–950 nm). Notably, the same species were found independently of the pH of the buffer at which the peptide was resuspended. At pH 7.4, a moderately higher number of medium-sized oligomers were detected, and after 1 h at RT, the TCP-25 solution was slightly hazy. Indeed, we identified large species with hydrodynamic diameters spanning 1 × 10^3^ – 5 × 10^3^ nm. Notably, similar results were also obtained when the sample was analyzed over extended times (up to 1 week). TCP-25 stored at pH 5 was completely limpid, and the oligomers detected at pH 5 after 1 h and up to 1 week were identical to freshly dissolved TCP-25. The fact that similar results were obtained when both samples were analyzed over time (from 1 h up to 1 week) indicates the rapid formation of stable equilibria at the different pH values.

The oligomers’ size distribution upon storage at 4 or –20 °C was also analyzed. The results at pH 5.0 showed a similar size distribution (Figure 5d), but the pH 7.4 sample contained large oligomers and aggregates of even larger size (Figure 5c, middle and last panels). However, the largest population disappeared after 15 min, and many medium-diameter oligomers appeared in their place. The same results were obtained after storing the samples at RT for 60 min. Interestingly, in the sample stored at –20 °C, we detected a high proportion of particles with small size, and their quantity decreased over time, indicating that the low temperature stabilizes them (Figure 5d, last panel). Taken together, the results provide further proof of the pH dependence of TCP-25 oligomer formation and give information about the influence of storage conditions on the stability of the formed oligomers.

### 3.6. Molecular Simulations of TCP-25 Oligomerization

To understand how TCP-25 forms oligomers, we used the 18-residue TCP, HVF18 [17], as a model peptide. Docking simulations using the NMR structure of HVF18 predicted that the peptide could potentially form a dimer in an anti-parallel fashion, but with only a limited number of inter-peptide contacts due to the “hooked” shape of the peptide. Thus, residue R4 on the loop of one subunit could interact with E18 on the C-terminus of the other subunit, with additional hydrophobic interactions between W8 and F16 at the dimeric interface (Figure S5a). Further docking indicated that the non-helical region of the peptide could form a potential interface for a tetrameric complex via hydrophobic interactions involving residues such as V2 and F3 (Figure S5b). Multiple tetramers would in turn be predicted to stack on top of each other to form higher-order oligomers (Figure S5c–d).

Atomic-resolution molecular dynamics (MD) simulations of the tetramer and octamer models were built using this NMR structure to assess their dynamics in solution and the likelihood that they may adopt conformations that are physiologically stable. However, the simulations suggested that the predicted oligomerization interface is likely to be unstable in solution. This is demonstrated by the high root-mean-square deviation (RMSD) values measured over timescales of hundreds of nanoseconds for the backbone of the peptides in each oligomer and the distortion of the tetrameric “building blocks” by the end of the simulations (Figure S6a).

**Figure 6:**
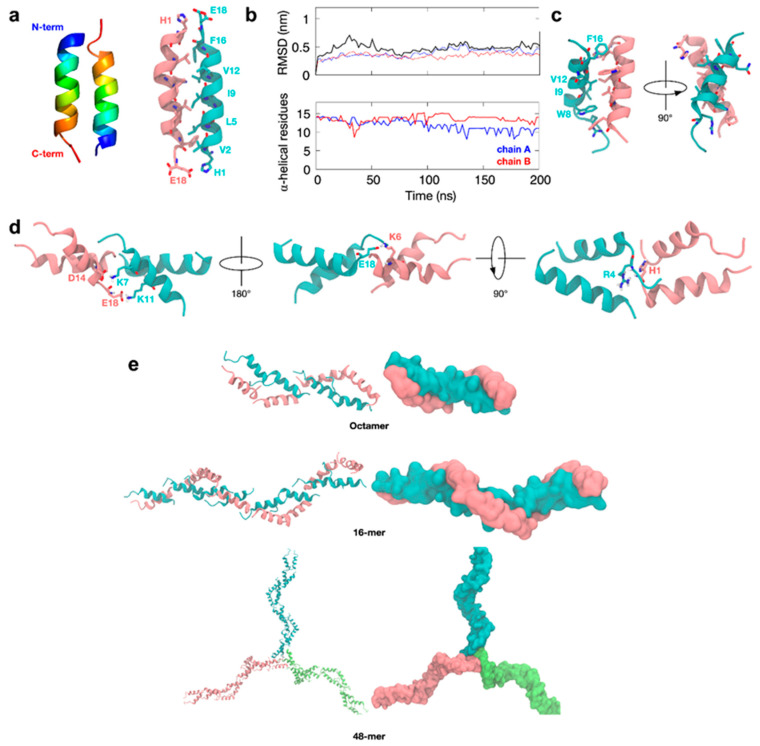
HVF18 idealized helix oligomerization. (**a**) A homology model of HVF18 was built as an ideal alpha-helical structure and the ClusPro program was used to generate a dimer. Two docking simulations were performed, and representative structures from the most populated clusters were aligned and colored in rainbow (left). Residues forming the dimer interface are highlighted in stick representation (right). (**b**) A 200-ns MD simulation was then performed, and the backbone root-mean-square deviation (RMSD) representing the structural drift during the simulation (top) was calculated for individual subunits (thin blue and red lines) and for the overall dimer (thick black line). The secondary structure preservation plot (bottom) shows the number of residues remaining in an alpha-helical conformation throughout the simulation for each chain. (**c**) Final image of the simulation showing residues that form the dimeric interface in stick representation. (**d**) Using the final snapshot as input for ClusPro, the structure of a tetramer was built. The representative structure of the most populated cluster is shown with the tetrameric interface highlighted in stick representation. (**e**) Octamer, 16-mer, and 48-mer structures were subsequently built using common oligomerization interfaces.

The “hook-like” NMR structure of HVF18 was solved while bound to LPS aggregates. It is possible that without LPS, the HVF18 peptide adopts a different conformation, such as a more extended alpha-helix. As such, we built a model of HVF18 based on an idealized, fully helical structure and then repeated the same docking protocol as described previously. Interestingly, the docking simulations again predicted an anti-parallel dimer for HVF18, similar to that based on the NMR structure. However, in this case, the key N-terminal H1 residue is expected to interact with the C-terminal E18, while a greater number of hydrophobic interactions (including V2, L5, I9, V12, and F16) would now stabilize the core of the dimer along the entire helical interface (Figure 6a).

Subsequent atomic-resolution MD simulations of this HVF dimer showed that the dimeric interface around the helical core remained stable, in contrast with the dimer based on the NMR structure. Furthermore, the two alpha-helices of the dimer tilted slightly with respect to each other and exhibited partial “fraying” of the termini to optimize interfacial interactions further (Figure 6b,c). To further test the likelihood of this dimer forming in solution, we next performed a series of microsecond timescale simulations at CG resolution, during which the monomeric HVF18 peptides were initially separated by at least 4 nm. In all cases, these peptides spontaneously reassembled into an anti-parallel helical dimer (Figure S7a,b), and all-atom simulations of this resultant dimer again showed it to be stable over hundreds of nanoseconds and to resemble the previously described models (Figure S7c).

Collectively, our results suggest a structurally reliable interface of the tilted, primarily alpha-helical anti-parallel dimer configuration. From these dimer models, we next built higher-order multimers using docking. The exposed termini of the dimer are oppositely charged, so they are predicted to form an interface for the formation of higher-order end-to-end oligomers. These dimers are predicted to be stabilized via interactions at acidic residues, including D14 and E18, as well as basic residues, such as K6, K7, and K11 (Figure 6d).

Interestingly, the key histidine H1 on the N-terminus of one dimer subunit is predicted to be within close proximity to R4 on another dimer subunit. Therefore, protonation at this histidine residue could compromise the stability of the interface, suggesting mechanistically how higher-order oligomerization may be dependent upon pH. Further docking simulations predicted that more dimer units could potentially be added to the ends to extend the existing structure into fibers, as well as form a center for micelle-like oligomers (Figure 6e) and hence extended networks.

Atomic-resolution MD simulations of resultant HVF18 tetramers, hexamers and octamers showed much lower RMSDs (Figure S6b) than the alternative multimers generated based on the NMR structure described above. This supports a more stable complex in this configuration. Furthermore, simulations mimicking the acidic conditions reflected by a protonated histidine resulted in partial disassembly of the oligomers (Figure S6c), which is consistent with the TEM results showing fewer oligomer particles at pH 5 (Figure 1d).

## 4. Discussion

There is increasing interest in developing HDPs as alternatives to conventional antibiotics and antiseptics. From this perspective, it is important to define the actions of the peptides at the molecular level for the rational development of new peptide-based anti-infective formulations. This would also help in understanding the behavior of formulated peptides, particularly at the often supraphysiological concentrations used for therapeutic applications. By defining the oligomerization behavior of TCP-25 and its prerequisites, the current study provides an explanation for the observed turbidity of the formulated TCP-25 hydrogel.

It is known that amphipathic peptides are inclined to form oligomers that are held together by noncovalent interactions and are in equilibrium with the monomer [42,48,49]. The organization of peptides in oligomers or aggregates is often associated with the induction of toxicity and immunogenicity, as well as a reduction in their activity [42,50,51,52,53]. However, for other groups of peptides, oligomerization or aggregation is an intrinsic part of the peptide’s natural mode of action. For example, recent evidence shows that oligomerization and supramolecular multimer formation are part of the mode of action of defensins and the cathelicidin LL-37 [54,55,56]. Therefore, oligomerization and aggregation can be compatible with peptide functionality. It is worth noting that the glucagon-like peptide-1 (GLP-1) receptor agonist liraglutide and the peptide hormone calcitonin are already on the market, whereas AMPs, such as LL-37, are in clinical trials [57,58,59,60,61]. In line with this, TCP-25 was found to oligomerize in a reversible manner, which is compatible with its observed efficacy in multiple in vitro and in vivo models [13,14,15,20].

In general, the environment has a strong influence on the organization of oligomers, which in turn affects their pharmacodynamic properties [62]. The sequence of TCP-25 contains a pH-responsive histidine residue, which is protonated at low pH. This makes the peptide more charged, with a change in net charge from +2 to +3 at low pH. This may lead to alterations in the amphipathic region and an increase in peptide solubility, leading to reduced oligomerization [63]. These results are reinforced by data showing that charged histidine has a low helix propensity [63].

Interestingly, previous studies have shown that protonation at pH 5.5 of this particular histidine residue also increases the antibacterial activity of TCP-25 against Gram-negative *Escherichia coli* by membrane disruption. Moreover, TCP-25 displays lower binding affinity to human CD14 when the pH is decreased, suggesting a switch in the mode of action from anti-inflammatory at neutral pH to antibacterial at acidic pH [64]. Together, these previous results and the present work demonstrate that a subtle protonation of the histidine residue in TCP-25 affects not only the activity but also the peptide conformation and oligomerization tendency.

CD analysis combined with DLS showed that a conformational change in TCP-25 accompanies peptide association and oligomerization. From a therapy perspective, an improved understanding of the oligomerization prerequisites and consequences should facilitate the preclinical and regulatory development of TCP-25. For example, it was demonstrated that oligomeric proteins are more stable and have higher T_m_ and C_m_ than monomeric proteins [65,66]. Analogously, TCP-25 was more resistant to both chemical and thermal denaturation under conditions that favored oligomerization. In particular, the thermal stability is very important since therapeutic peptides must withstand several processes during production, such as filtration and sterilization. Furthermore, they must be stored for a long time before they can be placed on the market. Accordingly, oligomerization could be exploited as a stabilizer of TCP-25 since the surface area will be smaller than in the monomer, so the peptide will be less prone to denaturation and protease cleavage [67].

It is also possible that oligomerization could facilitate a slower release of active molecules. For example, Pertinhez et al. showed that the synthetic peptide AKVTMTCSAS acts against *Candida albicans* only when it is in dimeric form. Furthermore, these dimers could also spontaneously and reversibly self-assemble in an organized network of fibril-like structures [68]. Gupta et al. provide another example in studies on the oligomerization of insulin at neutral pH using drug oligomerization as a drug depot. The active monomers were gradually released from the oligomers, but this release was dependent on the sequence and pH at which the oligomers were assembled [69].

The size range of TCP-25 oligomers is broad, but some sizes are more recurrent than the others, such as oligomers with hydrodynamic diameters of 0.46, 2.81, 4.58, 43, 230, 431, 462, 808, and 1740 nm. Furthermore, continuous change was observed in the sizes of the particles in solution, which indicates that the oligomerization of TCP-25 is a dynamic process that reaches an equilibrium with different lengths of time and buffer compositions. This flexibility of the peptide requires it to assume different conformations and form oligomers of different sizes, which may contribute to not only its stability, but also its activity and specificity, as reported for other proteins and AMPs [49,70,71].

## 5. Conclusions

With this study we have demonstrated that TCP-25 displays increase in α-helical structure and oligomerization at higher doses and neutral pH. TCP-25 is also more stable at higher concentrations when exposed to high temperatures or denaturing agents, which is compatible with oligomer formation. The studies on the formation of oligomers and its reversibility also showed that the process depends on pH, time, and temperature. From a pharmacodynamic and therapeutic perspective, this information is of relevance for the future development of TCP-25-based formulations and biomaterials where the peptides capacity to oligomerize could be exploited in order to control release dynamics, bioactivity, as well as degradation.

## Supplementary Materials

The following are available online at https://www.mdpi.com/2218-273X/10/11/1572/s1, Figure S1: Effect of pH on TCP-25 oligomerization, Figure S2: Thermal and chemical denaturation of TCP-25 at low concentration, Figure S3: Reversibility of thermal denaturation of TCP-25 at pH 5, Figure S4: Effect of pH and temperature on oligomerization of TCP-25, Figure S5: HVF18 NMR structure oligomerization interface, Figure S6: Atomistic simulation of HVF18 multimers, Figure S7: HVF18 dimerization interface from CG self-assembly.
