# Supplementary material for: Concentration- and pH-Dependent Oligomerization of the Thrombin-Derived C-Terminal Peptide TCP-25
Source: Biomolecules. 2020 Nov 19;10(11):1572. doi: 10.3390/biom10111572 (PMC7699335; doi:10.3390/biom10111572)
Supplement: Supplementary file 1 [file biomolecules-10-01572-s001.pdf]

Supplementary Figures

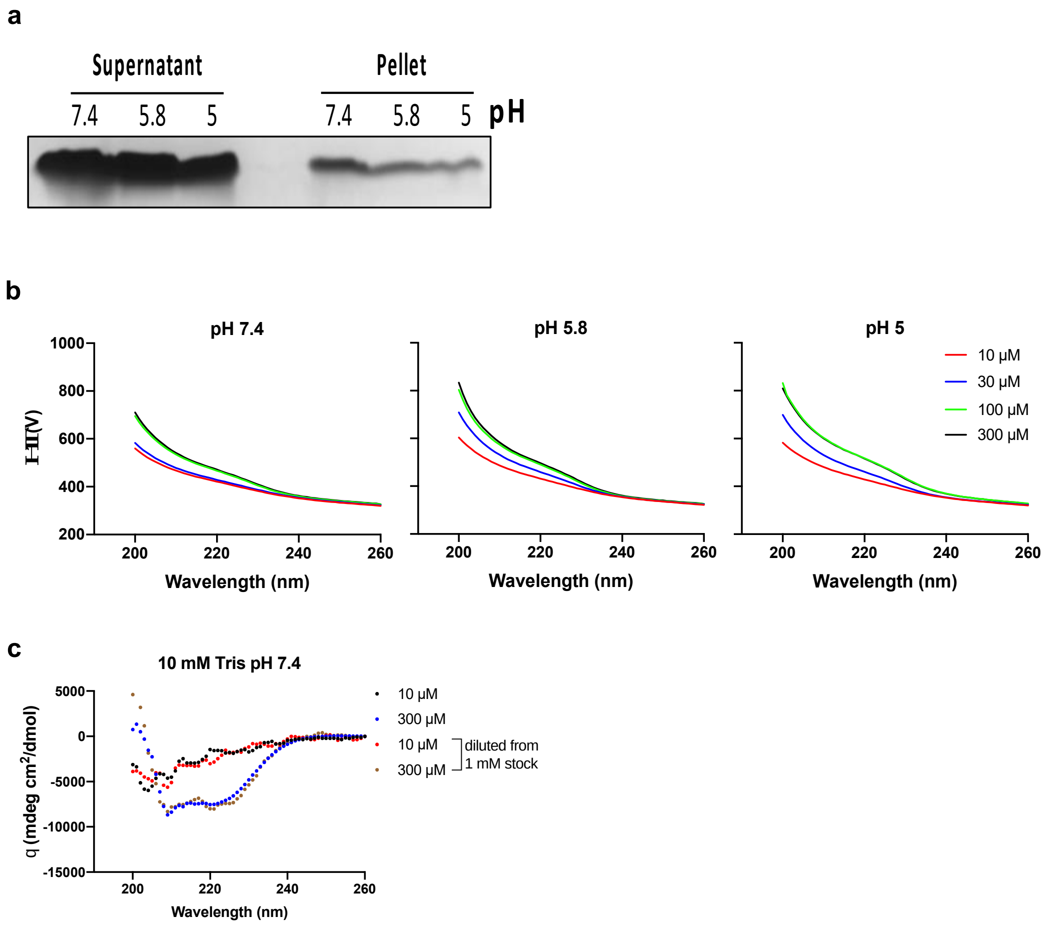

Figure S1. Effect of pH on TCP-25 oligomerization. (**a**) TCP-25 (300 µM) was dissolved in 10 mM Tris at pH 7.4 and centrifuged. The pellet was re-dissolved in 10 mM Tris at pH 7.4 or 10 mM NaOAc at pH 5.8 or 5.0. All samples were centrifuged again, and then the soluble and insoluble fractions were analyzed by SDS-PAGE. (**b**) Representative high tension (HT) signals of the CD spectra of TCP-25 dissolved at different concnetrations (10–300 µM) in 10 mM Tris at pH 7.4 or 10 mM NaOAc at pH 5.8 or pH 5 ($n = 3$). (**c**) Representative far-UV CD spectra of TCP-25 dissolved at 10 and 300 µM in 10 mM Tris at pH 7.4 or diluted at the same concentrations from 1 mM stock ($n = 3$). All the spectra were acquired at 25 °C.

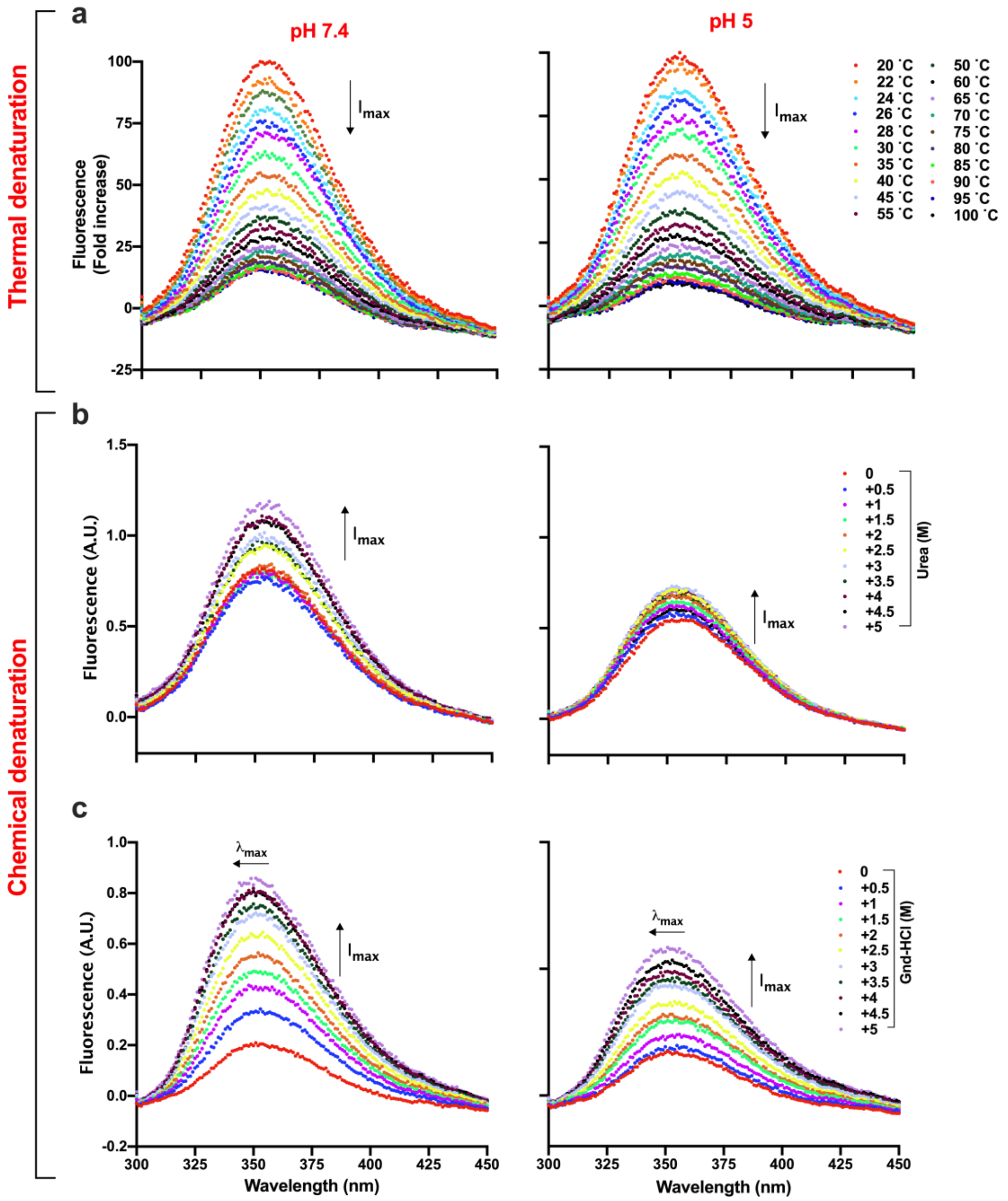

Figure S2. Thermal and chemical denaturation of TCP-25 at low concentration. TCP-25 (10 μM) in 10 mM Tris at pH 7.4 or in 10 mM NaOAc at pH 5.0 was denatured by increasing temperature (**a**) or by addition of increasing amounts of urea (**b**) or Gdn-HCl (**c**). The unfolding process was analyzed by recording emission spectra between 300 and 450 nm upon excitation at 280 nm. One representative emission spectra for each denaturation condition is shown (*n* = 3). ← indicates shift in maximum fluorescence intensity (λ_{max}); ↑I_{max} and ↓I_{max} indicate increase and decrease in maximum fluorescence intensity, respectively.

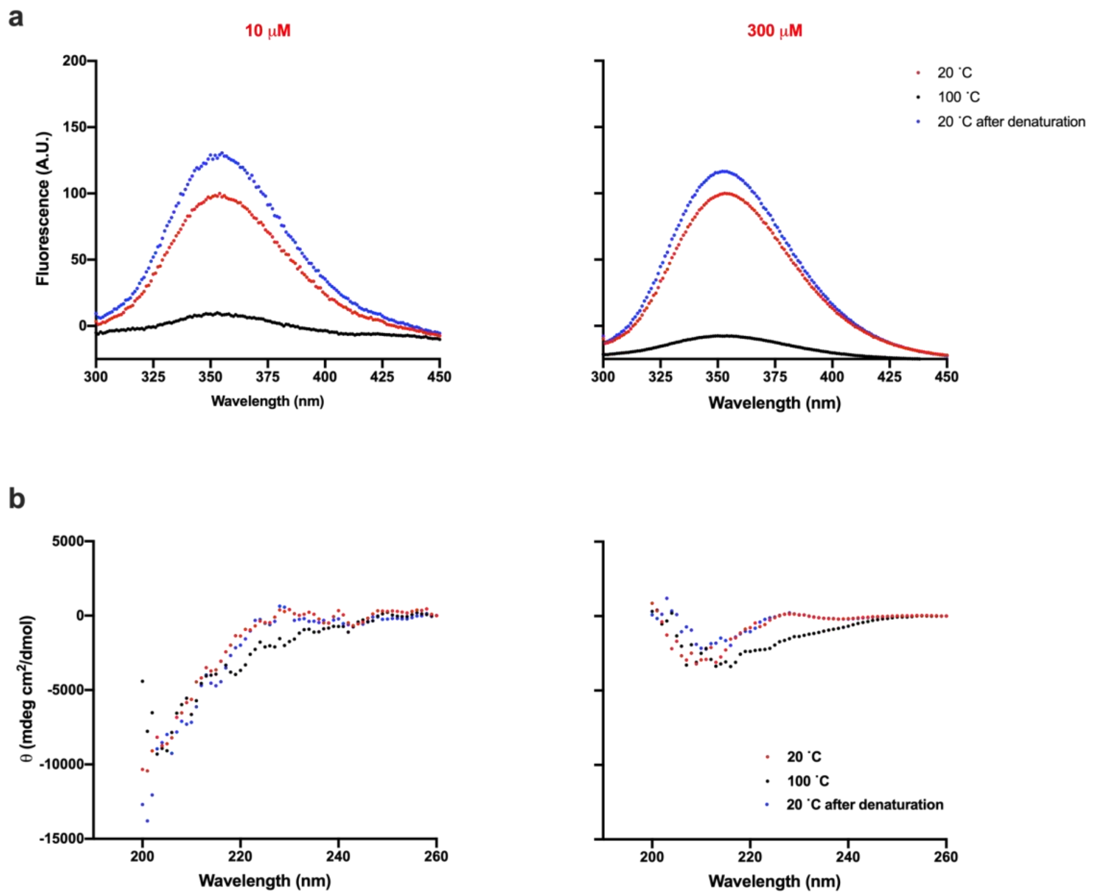

Figure S3. Reversibility of thermal denaturation of TCP-25 at pH 5. 10 and 300 μM TCP-25 in 10 mM NaOAc at pH 5.0 were denatured by exposing the peptide to 100 °C, and re-folding was analyzed by recording the intrinsic fluorescence of the peptide with a spectrofluorometer (**a**) or secondary structure with CD (**b**). The spectra were collected at 20 (red), 100 (black), and 20 °C after denaturation at 100 °C (blue). Each graph is a representative result of 3 independent experiments ($n = 3$).

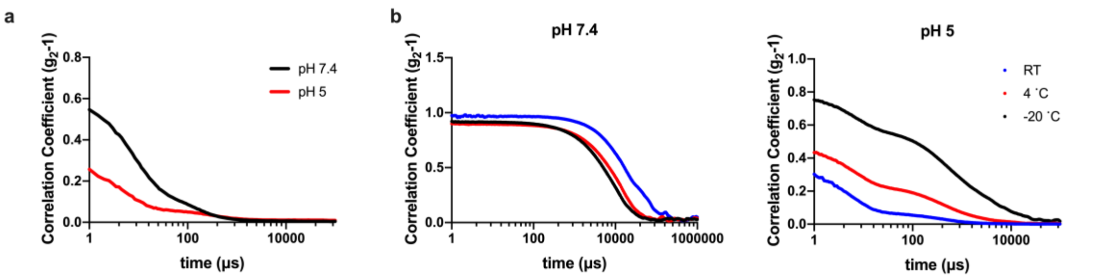

Figure S4. Effect of pH and temperature on oligomerization of TCP-25. (**a**–**b**) Representative correlation coefficient versus time in μs obtained from DLS analysis of 300 μM TCP-25 in 10 mM Tris at pH 7.4 or in 10 mM NaOAc at pH 5.0. Effects of pH (**a**) and temperature (**b**).

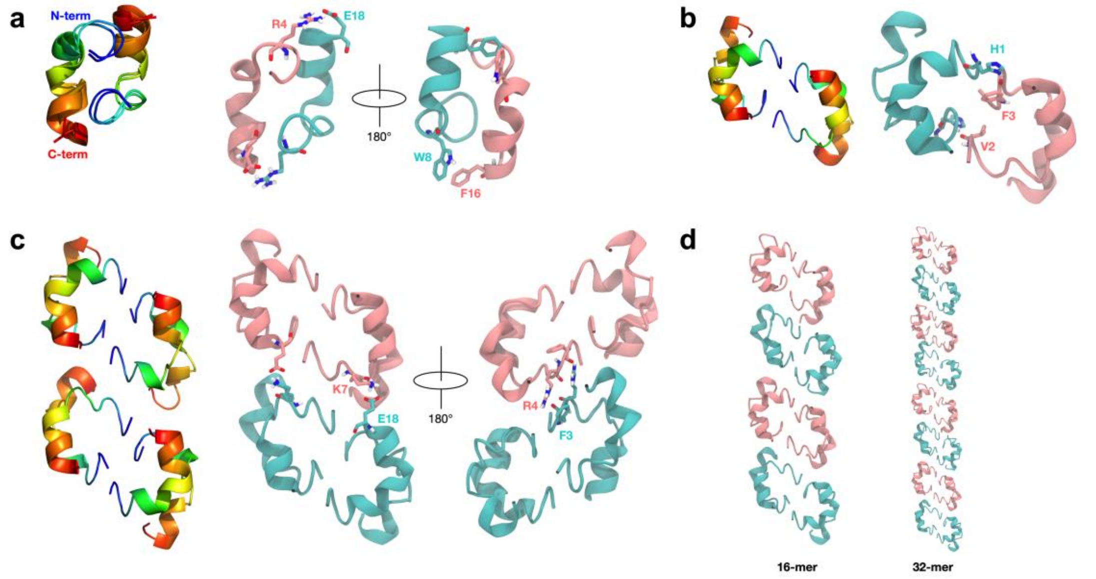

Figure S5. HVF18 NMR structure oligomerization interface. (**a**) HVF18 dimer generated using ClusPro protein-protein docking program using the NMR structure (PDB: 5Z5X). Two separate docking simulations were performed, and representative structures from the most populated clusters are shown on the left and colored in rainbow (from blue for N-terminus to red for C-terminus). Residues involved in forming the dimer interface are shown on the right. (**b**) The dimer was then used to generate a tetramer. A representative structure is shown on the left, and residues at the interface between dimers are shown on the right. (**c**) The tetramer was subsequently used to build an octamer. A representative structure is shown on the left, and the residues at the interface between the tetramers are shown on the right. (**d**) The

octamer was used to build a 16-mer (left) and 32-mer (right) with the same oligomeric interface.

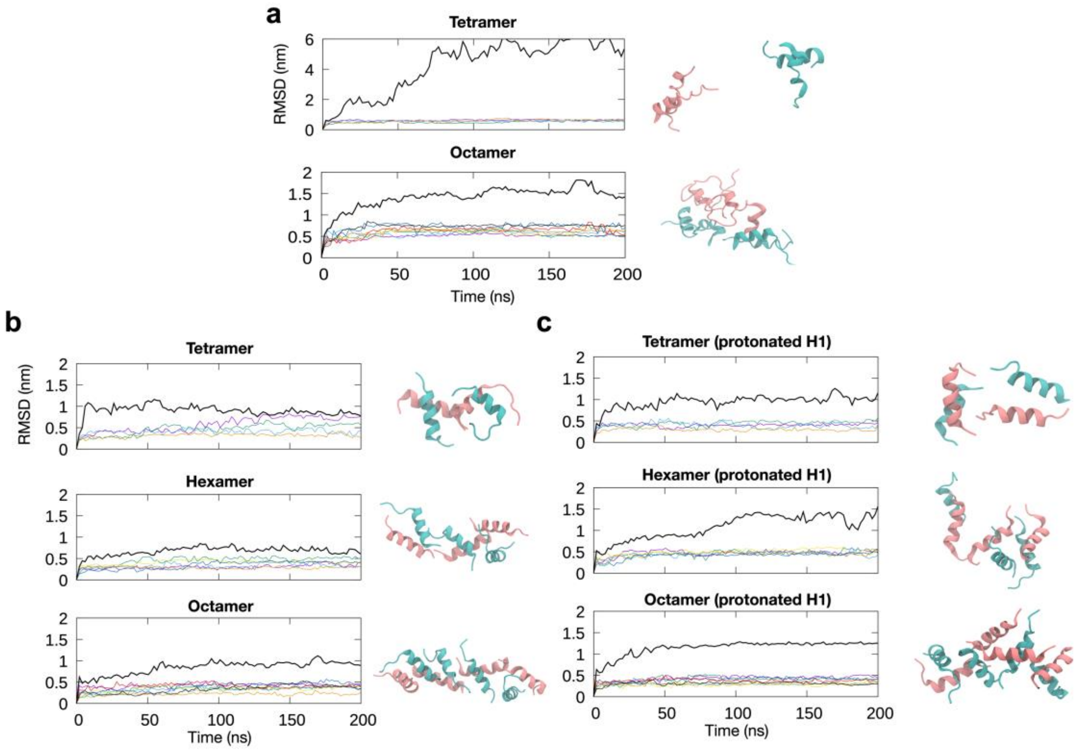

Figure S6. Atomistic simulation of HVF18 multimers. (**a**) (Left) Backbone RMSD of HVF18 peptides during 200 ns atomistic simulations of multimers generated by docking using the NMR structure (PDB:5Z5X). The thick black lines represent the RMSD of the multimer, whereas the thin lines represent the RMSD of individual HVF18 subunits. (Right) Images at the end of the simulations. For clarity, half of the peptide subunits are shown in pink, and the other half is shown in cyan. (**b–c**) Backbone RMSDs for simulations of HVF18 multimers generated by docking of an idealized helix model. The N-terminal H1 residue was neutral to mimic neutral pH conditions (**b**) and protonated to mimic low-pH conditions (**c**).

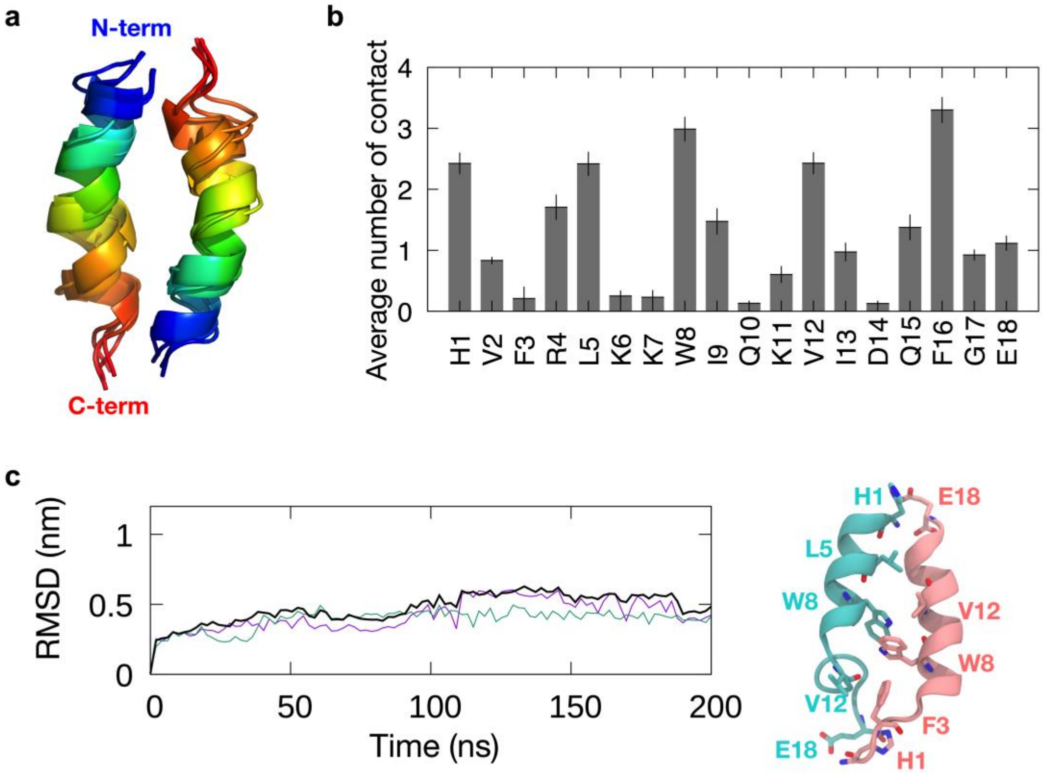

Figure S7. HVF18 dimerization interface from CG self-assembly. (**a**) Two copies of HVF18 homology model built as an idealized helix were placed far from one another and then simulated in solution at CG resolution. Five individual 1-μs simulations were performed. Representative structures from the most populated clusters from the last 0.5 μs were converted back to atomistic representation and overlaid, as shown in cartoon format. (**b**) Number of contacts made by each residue of one subunit with any residue in the other subunit from the last 0.5 μs averaged over the five simulations. Error bars show SD from the five simulations. (**c**) Atomistic simulation was performed using one of the representative dimer structures, and the backbone RMSD is shown on the left for individual chains (thin lines) and the whole dimer complex (thick black line). The final image of the protein is shown on the right with residues forming the dimer interface highlighted in stick representation.
